# A physical unclonable neutron sensor for nuclear arms control inspections

**DOI:** 10.1038/s41598-020-77459-3

**Published:** 2020-11-26

**Authors:** Sébastien Philippe, Francesco d’Errico

**Affiliations:** 1 Program on Science and Global Security, Princeton University, 221 Nassau St, 2nd floor, Princeton, NJ 08542 USA; 2 School of Engineering, University of Pisa and National Institute of Nuclear Physics, Pisa, 56100 Italy; 3 School of Medicine and Center for Emergency Preparedness and Disaster Response, Yale University, New Haven, CT 06520 USA

**Keywords:** Nuclear physics, Techniques and instrumentation

## Abstract

Classical sensor security relies on cryptographic algorithms executed on trusted hardware. This approach has significant shortcomings, however. Hardware can be manipulated, including below transistor level, and cryptographic keys are at risk of extraction attacks. A further weakness is that sensor media themselves are assumed to be trusted, and any authentication and encryption is done ex situ and a posteriori. Here we propose and demonstrate a different approach to sensor security that does not rely on classical cryptography and trusted electronics. We designed passive sensor media that inherently produce secure and trustworthy data, and whose honest and non-malicious nature can be easily established. As a proof-of-concept, we manufactured and characterized the properties of non-electronic, physical unclonable, optically complex media sensitive to neutrons for use in a high-security scenario: the inspection of a military facility to confirm the absence or presence of nuclear weapons and fissile materials.

## Introduction

Acquiring and sharing data that can be relied upon as honest or truthful is necessary for the economy, industries, and political institutions to function. Yet, in an age of digital disinformation and offensive cyber-operations, this has never been more challenging^1,2^. The problem is especially acute in national security issues such as the monitoring and verification of nuclear arms control agreements, where the ability to generate and act upon authentic, trustworthy, and accurate information about the nature and status of nuclear arsenals can help manage tensions, de-escalate crisis, and reduce the risks of nuclear weapon use.

An open challenge for arms control verification is how to acquire data that can be accepted as trustworthy by mutually distrustful parties^3^. So far, and whenever possible, states have collected their own data through nationally owned ground sensor stations, military satellites and other forms of intelligence collection. However, this approach places constrains on the scope and type of information that can be openly shared without revealing “sources and methods” to assess a state’s compliance with its treaty obligations. The approach also limits the possibility to collect certain kinds of data such as information about nuclear warheads and weapon-grade fissile materials inside sensitive facilities. Developing secure and trustworthy data acquisition systems that can be accepted as such by multiple distrustful parties could therefore broaden the scope of what can be verified, and by extension, the scope of what could be negotiated in future agreements^4–6^.

From a security point-of-view, this is an interesting case-study for four reasons: Attackers are states with virtually unlimited resources and access to state-of-the-art technologies, no common roots of trust exist between the participants, only agreed upon information can be revealed, and the stakes are unusually high. Yet in principle, designing secure sensors for arms control verification is not fundamentally different than for sensitive consumer or industrial applications: It requires demonstrating that the sensor data is authentic and truthful, and that the sensors themselves have not been compromised during their manufacturing by the addition of malicious functionalities, including the ability to manipulate data or secretly leak sensitive information that should not be revealed or even acquired in the first place.

To meet these requirements, traditional security and privacy solutions involve the use of cryptographic algorithms running on trusted hardware to authenticate and encrypt measured values a posteriori and outside the sensor media, and black-box tamper-indicating enclosures to limit physical access to critical components and information such as encryption keys^7^. Such an approach has well-known shortcomings. It presupposes that keys, algorithms, and unencrypted raw data are difficult to access, defeat, or modify externally and assumes a trusted supply-chain for the sensor hardware and software. However, raw data and encryption keys are at risk of side-channel attacks and extraction techniques^8,9^, and while enclosures may limit attacker access to critical hardware, they also prevent legitimate examination of what is happening inside and sent outside a device^10,11^. Unfortunately, even when complete access to a sensor electronic hardware is provided, the presence of embedded malicious functionalities, known as hardware trojans, cannot be completely ruled out^12,13^. Thus, for scenarios where no common root of trust or trusted third party manufacturer exist, the challenge of producing trustworthy sensor data remains open.

Here, we propose and demonstrate how to overcome these limitations using new passive, fully characterizable, and random sensor media that verifiably produce secure and trustworthy data in situ without relying on digital cryptographic algorithms, trusted electronics, and traditional tamper-indicating enclosures. The randomness of our sensor inherently and simultaneously provides a physical authentication and encryption mechanism for the measured data, and its passive, non-electronic, and non-digital nature makes these properties verifiable by anyone.

Our starting point is the established concept of physical unclonable functions (PUFs)^14,15^, and in particular, their non-electronic optical realization^16^. PUFs are randomly disordered, unique and unclonable physical systems, which generate complex outputs or “responses” when being excited by external stimuli or “challenges”. They are said to be “strong” when they possess a large number of distinct challenges—such that not all challenge-response pairs (CRPs) can be exhaustively measured in a feasible amount of time by an adversary—and when valid responses can only be elicited via direct measurement of the PUF^17^. This latter property implies resistance against numerical simulations of the challenge-response process or attempts at developing machine-learning models trained on a limited number of measured challenge-response pairs. An optical, strong PUF can be realized by probing a highly scattering medium with coherent light to generate light-field responses. By incrementally modifying the position or angle of incidence of the input light above a certain threshold corresponding to the limit of the optical memory effect^18^, it is possible to generate and record patterns that are uncorrelated to one another, unique to the media being probed, and hard to predict via simulation or modeling^19^.

As we show here for the first time, the responses of the optical PUF can also be made intentionally dependent on non-trivial physical stochastic effects such as exposure to low levels of ionizing radiations, in particular neutrons. Our approach is new with respect to other applications of PUFs to sensors in the sense that the strong PUF and sensing properties of our non-electronic media are inseparable and indistinguishable^20,21^. Below, we introduce our new sensor in the context of a nuclear arms control inspection, provide a protocol for its use, demonstrate experimentally its key properties, and discuss its security. Overall, our findings open a new avenue of research in the application of non-electronic physical unclonable functions to verifiably honest and secure sensing.

## Results

### Sensor properties and measurement protocol

**Figure 1:**
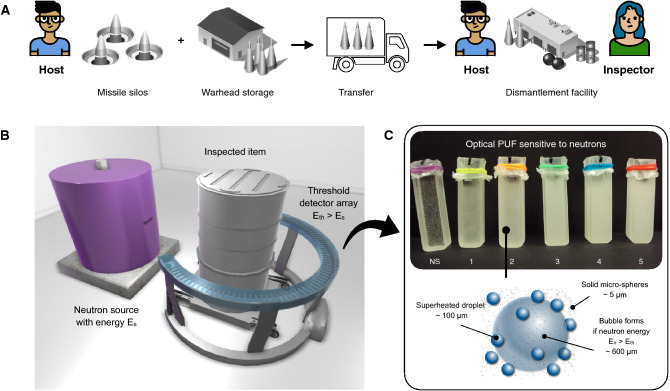
Inspecting for the presence or absence of fissile materials with optical physical unclonable neutron detectors. (**A**) The Host removes nuclear missiles from operation and dismantles the associated nuclear warheads in a specific facility on its territory. The Inspector monitors the operations. (**B**) As part of verification procedures, inspected items are tested for the presence or absence of fissile materials at various stages of the dismantlement process. To do so, items are placed between a neutron source of energy E_s_ and a neutron detector array with detection threshold E_th_ > E_s_. In this situation, only if fissile materials are present in the item can the detectors record the presence of neutrons with energy E_n_ above E_th_. (**C**) The detectors’ sensor medium is optically complex. It comprises superheated droplets and inert microspheres dispersed in a viscous gel matrix. Upon interaction with a neutron of energy E_n _> E_th_, droplets can vaporize and expand into macroscopic bubbles, irreversibly and unclonably modifying the internal spatial distribution of drops and microspheres.

As part of a hypothetical nuclear-arms reduction agreement, state A (the weapons host) has committed to removing a number of nuclear missiles from operation and dismantling the associated nuclear warheads in a specific and access-restricted facility on its own territory (Fig. 1). State B (the inspector) requests to monitor treaty compliance by regular (or random, short-notice) inspections, including sensor installation and measurements at State A’s site. One simple and valuable measurement that both parties are considering consists in testing objects (e.g. containers) or locations (e.g. dismantlement bays or temporary storage vaults) for the presence or absence of fissile isotopes, uranium-235 and plutonium-239, the key ingredients for nuclear weapons. This would allow monitoring the flow of materials inside the dismantlement facility, as well as any authorized shipment leaving the premises^22^.

To be accepted by both parties, the sensors fabricated by B must meet the following requirements^3^: First, B must demonstrate to A that B’s sensors do not possess any hidden capabilities or remotely actionable functionalities that could compromise the safety of A’s nuclear weapons or personnel or perform covert measurements. Second, A must not be able to replace or alter sensors provided by B in order to compromise sensor data reported to B.

We met these requirements by designing a sensor that is both an optical physical unclonable function and built from materials that passively, randomly and irreversibly change their physical properties upon neutron exposure.

As shown in Fig. 1, our novel optical PUF sensor comprises two types of scatterrers: 100 μm superheated fluorocarbon drops and 5 μm solid microspheres suspended in a thick, inert and immiscible gel matrix. The drops can expand into stable bubbles of diameter ~ 600 μm when exposed to neutrons with energy E_n_ above E_th_ corresponding to the neutron energy threshold required to trigger vaporization^23^. Both the threshold energy and sensitivity of the detector can be selected by using different emulsified halocarbons and drop sizes, respectively. The microspheres are used to enhance the overall light scattering properties of the medium without affecting the functionality of the sensor. The expansion of drops into bubbles displaces scattering centers in their vicinity, permanently affecting the transport of coherent light inside the PUF sensor media.

**Figure 2:**
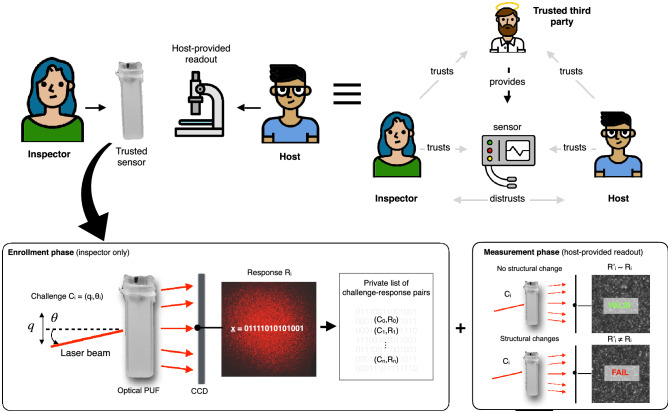
Sensor challenge-response protocol. An optical PUF sensor measurement protocol emulates the properties of a trusted third-party protocol *in the absence* of a trusted third party. The sensor is provided by the inspector party, who enrolls it privately before bringing it to the host-controlled facility. The enrollment phase consists in creating a private list of challenge-response pairs by probing the sensor media with coherent light at different position and angle of incidence and recording the output light fields. The outputs are converted to bit strings to facilitate their comparison and improve reproducibility. Once detectors are enrolled, the protocol does not require a trusted read-out for the measurement phase. Once the inspection measurement is over, the detectors are probed again, and responses are compared to previously recorded values. If they match the record, the inspector confirms that the detectors structures have not changed, and in our case that the sensor were not exposed to neutrons with energy E_n_ > E_th_.

Notably, this property implies that as long as the light scattering behavior of the sensor does not change, one can rule out its exposure to neutrons with energy above E_th_. This fact can be checked in a simple challenge-response protocol between State A and State B, using a list of challenge-response pairs collected during the PUF’s private enrollment stage on B’s side for comparison during the measurement phase (Fig. 2). An important advantage of this approach is that the electronic equipment for reading out the sensor media (and for communicating its responses) does not need to be trusted by the inspector and can be provided by the host. Instead, the unclonability, uniqueness, complexity, and verifiability of the sensor alone suffice as trust anchor and for establishing security.

A proof-protocol to demonstrate that the detectors were not irradiated could be conducted as follow:

#### Setup phase (enrollment): inspector only

1. The inspector manufactures an optically complex superheated emulsion detector.
2. The inspector determines a private Challenge Response Pairs-list L for the detector (Fig. 2). For *i* = 1, … ,n, she randomly chooses challenges C_i_ = (q_i_, θ_i_), directs a laser beam at coordinate q_i_ with angle θ_i_, and measures the resulting optical responses R_i_ from the detector media.
3. The inspector repeats steps 1 and 2 of the enrollment procedure for each detector. The inspector then brings the detectors to the host-controlled facility.

#### Validation phase: host only

The host can check non-destructively the detectors it received via standard measurement practices to verify for example that they do not contain explosive materials or forbidden chemicals. This can be done through standard X-ray irradiation (with the benefits that our detectors are insensitive to photons). Additionally, the host can select a subset of detectors randomly for irradiation and destructive assay. Note that detector functions can also be verified destructively after the inspection is completed.

#### Proof phase (standard non-irradiation): inspector and host

1. The host claims that the detector internal structure has not been modified, including through exposure to neutrons with energy E_n_ above the detector detection threshold E_th_.
2. For *v* = 1, … , m, with m < n the inspector randomly selects (C_*v*_, R_*v*_) pairs and sends C_*v*_ = (q_*v*_, θ_*v*_) to the host. For each C_*v*_ the host directs the laser beam to the PUF according to (q_*v*_, θ_*v*_) and provides the resulting optical response R_v_’ back to the inspector.
3. If all R_v_’ ~ R_v_, the inspector accepts the proof. She then removes the (C_*v*_, R_*v*_) pairs from the list L.

Here the soundness and completeness of the protocol are based on the assumption that, for any host tampering-strategy following the set-up phase (e.g. the modification or swapping of detectors) and for each detector, there exists at least one challenge v* such that the response R_v_*’ ≠ R_v_*. Formally, we assume that if a detector has not been modified, then it passes the proof phase test for all challenges with probability of at least (1 − α), where α is the false-positive rate. Furthermore, we make the physical assumption that there exists a value β such that for any two detectors D and D’, if D structurally differs from D’, then there exists a challenge such that testing with these parameters the inspector will accidentally declare D’ to pass the inspection with probability of at most β. For classical optical PUF, β is extremely small since minute structural differences—for example, the displacement of just a few scattering centers from their reference positions – are sufficient to produce different responses^24^.

With such properties, these sensors are particularly suitable for active neutron or photon interrogation measurements to check for the presence or absence of fissile isotopes in sensitive items (nuclear weapons or sub-assemblies thereof)^25,26^.

**Figure 3:**
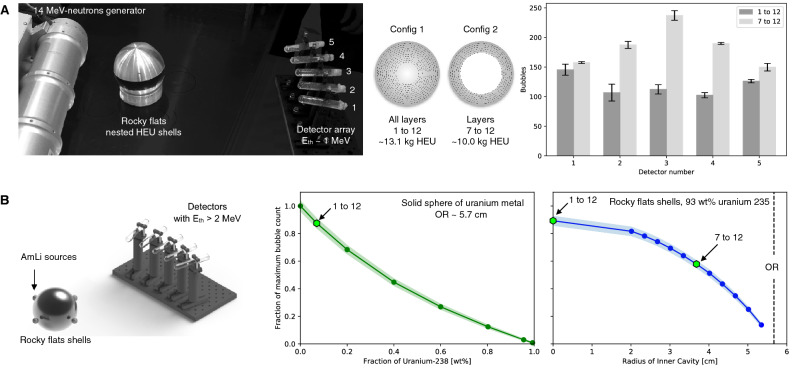
Information available from the active interrogation of highly enriched uranium objects with superheated emulsion detectors. (**A**) Transmission measurements (E_th_ < E_s_) of the Rocky flats highly enriched uranium nested shells (93.5% uranium-235) were performed with superheated emulsion detectors and a compact 14-MeV neutron source in 2-min irradiations at the US Nevada National Security Site Device Assemble Facility^27^. The obtained radiographs (bar chart) could reveal sensitive geometric and neutron opacity information. (**B**) Monte Carlo simulations of driven emission (also called active interrogation) measurements (E_th_ > E_s_) show that two spherical uranium metal objects of identical outer radii, a solid sphere with a fraction x_238_ of uranium-238 (green curve) and a thick shell with fraction x’_238_ < x_238_ (blue curve), could produce identical bubble counts when exposed in the same configuration.

To this purpose, the previous protocol can be extended to demonstrate that an object does not contain fissile materials, without revealing any other information:

1. The object is placed between a neutron source of energy E_s_ and an array of detectors with neutron threshold energy E_th_ such that E_th_ > E_s_ (Fig. 1).
2. The source energy and fluence are monitored by both parties independently. For inspectors, this can be done using non-electronic monitor tags. It is important for the host that E_s_ < E_th_ to avoid the release of sensitive information through transmission measurements (Fig. 3A).
3. After the source is turned off, the detectors are removed from the array and randomly scrambled. This step prevents the host from introducing dummy detectors before the inspection takes place, while keeping genuinely enrolled detectors for challenge-response measurements in a separate location.
4. Both parties then check whether the detectors were modified through the appearance of bubbles. To do so, the host and inspector check the status of the detectors following the challenge-response protocol described previously.
5. If no detector recorded bubbles, the inspector accepts the proof of fissile material absence.

Here the protocol soundness relies on the fact that only the presence of fissile materials can generate (fission) neutrons with energy higher than the neutron detection threshold (since E_s_ < E_th_). A similar proof can also be achieved with a high energy source of X-rays inducing photo-fission in fissile isotopes^28^. Because the detectors are insensitive to photons (X-rays and gammas included), the requirement on the energy threshold derives from the need to avoid the detection of photo-neutrons from naturally occurring deuterium; these neutrons can reach 3-MeV when produced by 9-MeV X-rays^29^.

Finally, from a privacy point of view, our protocol is essentially zero-knowledge^30,31^. It does not reveal information about the quantity, configuration, and isotopic composition of objects presented to the inspector beyond confirming the absence or presence of fissile materials. This is the case because: no transmission information is recorded and different fissile objects can produce identical bubble counts (Fig. 3B).

### Experimental realization with optically complex superheated emulsions

To validate our approach, we conducted a proof-of-principle study including sensor fabrication and characterization. To start with, we filled small optical glass vials with neutron-sensitive superheated emulsions of fluorocarbon droplets (see “Methods”). These droplets were introduced and distributed randomly in an immiscible and viscous water-based polymer-gel matrix at a concentration of around ~ 4000 per cm^3^. In addition, we doped the matrix gel with a large number of smaller micron-size inert microspheres at concentrations of up to ~ 7. 10^7^ per cm^3^, such that the resulting medium was both functional and optically complex. The drops, microspheres, and matrix densities were closely matched to provide a basis for reproducible measurements.

To verify the optical PUF properties of our sensors, we characterized the size of their challenge space, and tested whether the measured challenge-response pairs prior and subsequent to neutron exposure were indeed different and decorrelated. We did so by first probing unexposed detectors at different laser positions and comparing far-field output interference patterns recorded with a digital camera to the output at a reference position. We then exposed our emulsions to neutrons with energy higher than the sensor detection threshold, while recording the output light field for a given input position.

**Figure 4:**
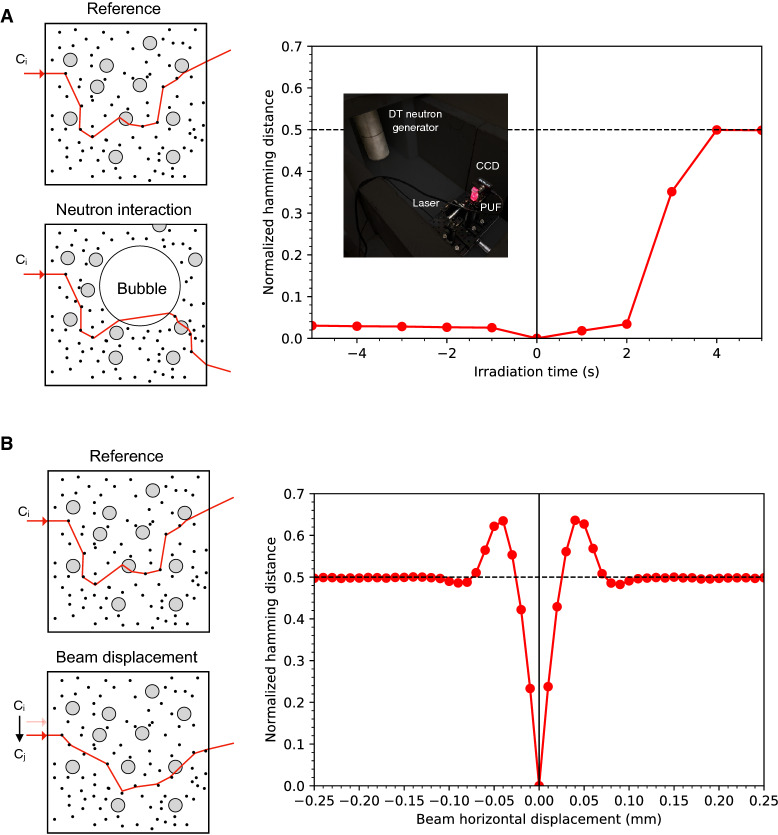
Decorrelation of an optical PUF sensor response via neutron interaction and laser beam displacement. (**A**) Experimental data show the decorrelation effect of neutron irradiation on previously recorded response. The time series is obtained by comparing responses to a given challenge at each time t = t_i_ to the reference response at t = 0, the time at which the neutron generator is turned on. The image insert shows the experimental apparatus. (**B**) Experimental data show the decorrelation effect of laser beam displacement on the sensor surface.

To compare responses, we processed the recorded images with a 2D Gabor transform-based error correction code to reduce the effect of pixel noise, compensate for mechanical misalignments, and generate reproducible bit strings (see “Methods”). The correlation between response strings was then measured through their normalized Hamming distance (HD), defined as the number of bits that are different at each position along two binary strings normalized by the string length. When strings of identical length are sampled from two independent random variables, their average Hamming distance is 0.5. Our experimental results (Fig. 4) confirm the decorrelation effect of neutron irradiation on previously recorded response as well as the sensitivity of responses to small displacements of the laser beam on the sensor surface.

We used the latter result to estimate the size of our challenge space. Accounting for the possibility to probe each possible laser position q on the sensor surface at different angles of incidence sampled within a Δθ = π/2 solid angle and spaced by δθ = λ/2πL ~ 10^–5^ corresponding to the optical memory limit of the media, the number of individual challenges available for a single sensor is given by (2A/δq^2^)(Δθ/δθ)^2^ with A and L being the detector surface and thickness respectively. Our measurements show that the average decorrelation of the output field happens after transverse displacements of δq ~ 0.025 mm (Fig. 4B). Thus, for each one of our sensors, there are about 2.5 10^16^ challenges from which different and uncorrelated responses can be recorded. To defeat our PUF by measuring all the possible challenge-response pairs, an attacker would therefore need about a year assuming he or she can measure one CRP every nanosecond without being interrupted. Because we expect our detectors to be in the custody of inspectors until they arrive on site and assume that inspections would take days (perhaps weeks) at most, our approach can be considered robust against such an attack.

**Figure 5:**
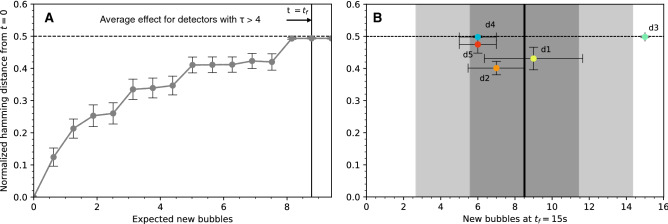
Neutron decorrelation results for detectors with increasing optical complexity. (**A**) Average evolution of Hamming distance for detectors with optical depth τ > 4 (type 3, 4, and 5) (**B**) Normalized hamming distance between the transmission patterns at t = 0 s and at t = 15 s corresponding to a single challenge, as a function of the number of new bubbles. Error bars are 1 s.e.m. The vertical line at b = 8.505 is the expected number of bubbles at t = 15 s and the dark and light grey regions corresponds to 1-sigma and 2-sigma deviations respectively.

To study the sensitivity of neutron-induced response decorrelation to photon mean free path in the sensor media, we produced five types of emulsions with increasing optical depth τ (see properties in Extended Data Table 1) and exposed them to neutrons (Extended Data Fig. 1). For a given challenge, our results presented in Fig. 5 show that the corresponding response becomes fully decorrelated (HD = 0.5) after the appearance of as little as one and as many as 9 bubbles for detectors with τ > 4 (corresponding to 16 scattering events per photon between input and output fields).

Finally, we used our results to compute the intra distance (valid challenge for a given detector) and inter distance (given challenge on different detectors) distributions for our sensors (Extended Data Fig. 2). The results, typical for an optical PUF, support the uniqueness of each sensor.

In future iterations of our sensors, we envision that smaller sub-micrometer microspheres and drops at potentially higher concentration could be used to achieve even higher degrees of structural and optical complexity^32^. Yet, the entropy of our current neutron-sensitive media already exceeds the original optical PUF design, which employed spheres of diameters ~ 650 μm with density ~ 1400 per cm^3^, by several orders of magnitude^16^.

### Security considerations

To defeat our sensor, and other optical PUFs in general, an attacker needs to either produce an exact copy or gain the capability to predict its responses through simulations.

Cloning attacks would require perfect knowledge of the positions, shapes, and sizes of all microspheres, as well as high-precision controlled manufacturing techniques to reproduce this architecture. While very high resolution 3D scans of the sensors could be obtained with modern micro- or nano-X-ray computed tomography or magnetic resonance imaging techniques^33,34^ , manufacturing an exact clone would still be extremely difficult, if not impossible. The emulsification of a metastable superheated liquid in another fluid is a complex process. Magnetic stirrers, ultrasound fractionation, or coaxial flow techniques are employed^35^ and have no known equivalent in 3D precision manufacturing and printing. Because the injection and mixing steps are stochastic in nature, they randomly influence the fluorocarbon droplet sizes and the locations of all scatterers (up to ~ 10^8^ per cm^3^ for our detectors). Each sensor is thus unique and impossible to reproduce exactly even by the original manufacturer.

With regards to predicting PUF responses, we consider two types of attacks: numerical simulations and machine-learning models.

Assuming that the 3D internal structure of each sensor is perfectly known, the complexity of numerically computing a PUF-sensor’s responses would still be overwhelming for adversaries: prior studies estimate that in the case where every cubic PUF-subpart with an edge length equal to the wavelength of the probing laser influence the optical response, around 10^26^ computing operations would be necessary to emulate an optical PUF of size 1 cm^3^. Modern supercomputers have yet to break the exascale barrier^36^, but assuming they will, adversaries would need about 3 years per cm^3^ of sensor media to simulate the response of a given challenge. This is far from the few seconds that are required for breaking our protocol by providing correct responses in a reasonable amount of time.

Perhaps more threatening than brute force computations are modeling attacks leveraging machine learning algorithms trained on a subset of measured challenge-response pairs^37^. To the best of our knowledge, however, no such attack has yet been successfully demonstrated against optical PUFs. Recent advances in imaging through optically complex media, via measurements of transmission matrices^38^ or the application of deep learning techniques to this problem^39^, provide an interesting avenue for developing modeling attacks. Fortunately for optical PUFs, these approaches are still unable to predict the scattering behavior of complex media above the limit set by the optical memory effect that physically defines the boundaries of individual challenges^40^.

Thus, the transport of coherent light in our sensors, and optical strong PUFs in general, continues to provide a unique “fingerprint” of their internal structure that is hard to forge physically or digitally even for a resourceful and sophisticated attacker employing state-of-the-art techniques.

## Discussion

We have established new principles to design verifiably secure sensors that do not rely on classical cryptographic algorithms and trusted read-out equipment to function, addressing important shortcomings of classical security and privacy approaches to the acquisition and sharing of sensor data. As an example, we manufactured and demonstrated key properties of passive, physically and digitally unclonable, optically complex media that are sensitive to neutrons and designed for use in nuclear arms-control inspections.

The resulting sensor media derives its security from its fully-characterizable, non-electronic, and random nature. The complex unclonable and hard-to-predict outputs of the sensor automatically authenticate any measured values, avoiding the need for any cryptographic post-processing. The fully-characterizable, non-electronic nature of the sensor provides a way to verify the absence of malicious functionalities. These properties allow us to decouple the security and privacy requirement for reading and communicating sensor response from the sensor media itself, and at the same time, overcome limitations of trust models between sensor manufacturers and users, which have been a fundamental challenge of arms control verification.

While our approach leverages the properties of the classical optical strong PUF, interesting alternative approaches could be developed leveraging quantum one-way functions^41^ and quantum secure communication protocols^42–44^ with the goal of developing quantum mechanically secure and unclonable sensors for radiation measurements.

For arms-control verification, our results provide an appealing solution to the long-standing challenge of authenticating and certifying inspection equipment, potentially removing a major technical obstacle in supplying inspector-provided apparatus to perform measurements in sensitive facilities. Demonstrating the viability of this approach in actual exercises and through red teaming by government experts could expand the scope of future bilateral and multilateral arms-control verification seeking to place and verify limitations on all warheads.

## Methods

### Detector characteristics

Our detectors comprise standard 10 mm square spectrophotometer cuvettes (1 × 1 × 3 cm^3^ volume) filled with an emulsion of octafluorocyclobutane, C_4_F_8_, with 100 µm diameter droplets (average drop density of 4000 per cm^3^) and 5.2 ± 0.42 µm diameter spheres (PS06N Bang Laboratories) of different concentrations (from 8.7 to 69.5 × 10^6^ per cm^3^) dispersed in a viscous aqueous gel matrix. They have an absolute efficiency of ~ 2.25 × 10^–4^ bubbles per crossing 14-MeV neutron in the detector volume. Their optical properties including absorbance A, transmittance T = 10^−A^, optical depth τ = − ln(T), and transport mean free path l* = L/τ (with L the cuvettes' width) are summarized in Extended Data Table 1^45^. Assuming that the scattering path results from random walks, photons scatter N = (L/l*)^3^ times on average. For the most turbid detectors shown in Extended Data Fig. 1A, N ~ 29 for τ ~ 5.4. Other particles and microspheres were also investigated including zinc-oxide (ZnO) and silica (SiO_2_). These did not disperse well in the gel matrix, reacted with the fluorocarbons or did not withstand detector recompression at 70 atm (the pressure used to re-condense bubbles in a detector). The long-term stability of the viscous gel matrix was the subject of a prior study (albeit without the presence of solid microspheres)^30^. Stability was evaluated in terms of bubble displacement and growth following exposure. No change was discernable for the first two months. Other manufacturing techniques including the use of a stiff polymer as matrix material^46^ could possibly enhance pre-irradiation stability (albeit at the expense of reusability and long-term post irradiation stability) depending on the use case scenario.

### Experimental apparatus

The beam from a compact laser diode with center wavelength λ of 635 nm and diameter φ of 2.9 mm is directed onto a superheated emulsion detector held by a mount capable of fine transverse motion. A 1280 × 1024 pixels CMOS monochrome sensor (Thorlabs DCC1545M with 5.2 µm square pixels) collects images resulting from the interaction of the laser with the detector. The apparatus is placed in a shielded irradiation canal. The detectors are exposed to 14-MeV neutron from a Thermofisher P-385 DT neutron generator. The resulting data are processed through a Gaussian pyramidal transform (gaussian blur and subsampling technique) implemented in sequence with a Gabor transform (similar to a Fourier transform used in features detection), as described in Pappu’s PhD thesis^47^. Both functions are available through the scikit-image Python collection^48^. The algorithm help stabilize the reproducibility of the response generation process by converting the output images into strings of 2^(19−2n)^ bits with n representing the Gaussian pyramidal level.

## Supplementary information

Supplementary Information.

## Data Availability

All data needed to evaluate the conclusions in the paper are present in the paper and/or the Supplementary Materials.
