# Supplementary material for: A physical unclonable neutron sensor for nuclear arms control inspections
Source: Sci Rep. 2020 Nov 26;10:20605. doi: 10.1038/s41598-020-77459-3 (PMC7692483; doi:10.1038/s41598-020-77459-3)
Supplement: Supplementary file 1 — Supplementary Information. [file 41598_2020_77459_MOESM1_ESM.pdf]

A Physical Unclonable Neutron Sensor for Nuclear Arms Control Inspections

Sébastien Philippe,[1]* Francesco d'Errico,[2,3,4]

[1] Program on Science and Global Security, Princeton University, 221 Nassau St, 2nd floor, Princeton, NJ 08542, USA
[2] Yale Cancer Center, Radiology & Biomedical Imaging, Yale University, PO Box 208042, Tompkin's East 2, New Haven, CT, 06520-8042, USA
[3] Instituto Nazionale di Fisica Nucleare, Edificio C, Largo Bruno Pontecorvo, 3, 56127 Pisa PI, Italy
[4] Universita di Pisa, Scuola di Ingegneria, Largo Lazzarino 1, 56126 Pisa, Italy

Supplementary Materials

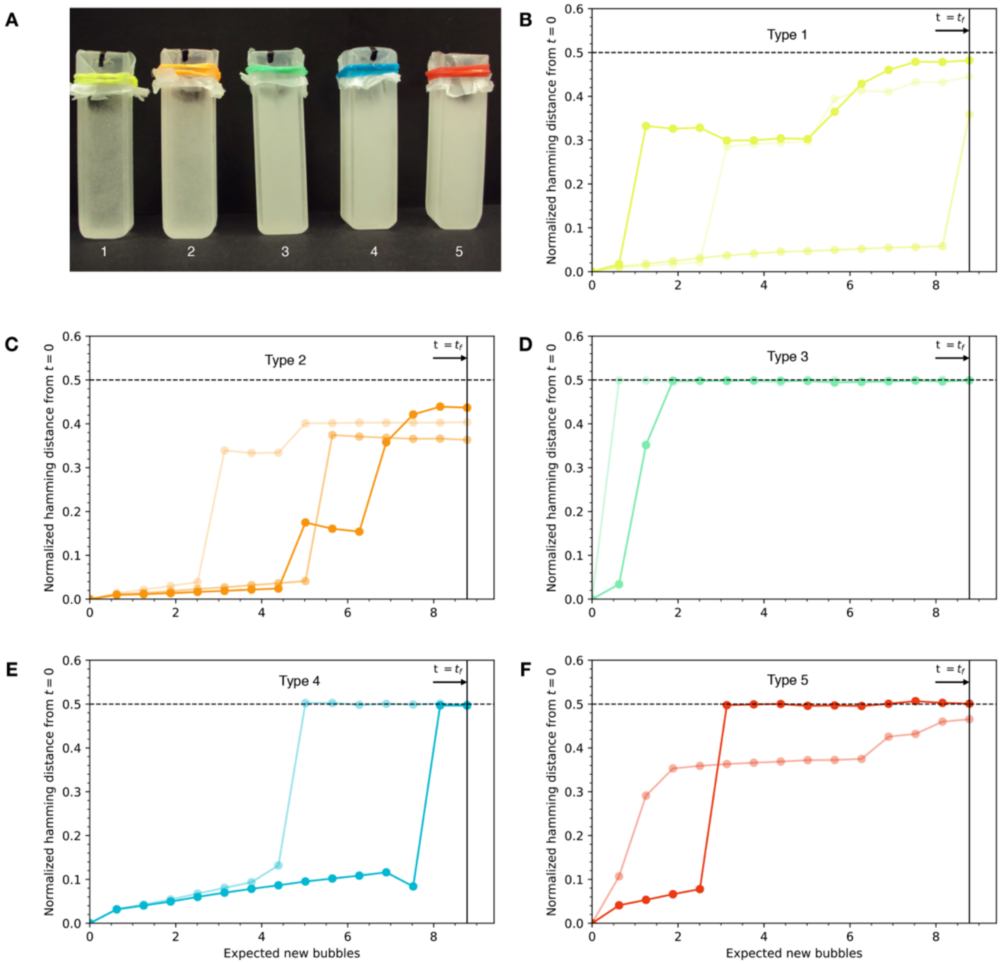

Extended Data Fig. 1. Experimental results for multiple neutron detector-exposures. Evolution of the hamming distance from t=0 to t=t_f as a function of expected number of new bubbles when detectors (**A**) are exposed to a neutron flux of ~920 neutron s^{-1} cm^{-2}. Figures (**B**) to (**F**) correspond to detectors type 1 to 5 of increasing optical depth τ (each curve is a single detector-run). In some cases, neutron induced decorrelation is extremely rapid, happening after the appearance of 1 bubble; in some cases, it requires up to ~8 bubbles. These numbers correspond to 0.6% and 5% of the maximum detector capacity (assumed to be of the order of 150 bubbles for a ~3 cm3 counting volume). The distance between the nucleation sites and the input laser beam position along the z-axis of the detector explains this difference. If a bubble appears at a position z_b relatively close to the input laser beam position z_i, it will have a dramatic effect on existing transmission channels. (For 3D multiple-scattering media, the displacement of a small number of scatterers is enough) The further away a bubble appears, the less effect it may have. In the limit where $0< z_b<<z_i$, the contribution of a bubble is to displace the gel matrix upwards by ∂z~0.001 mm. If the bubble appears at $z_i<< z_b<z_{max}$, the bubble has a limited effect because the detector is open ended at $z = z_{max}$. By probing the detector at multiple positions, the chances of detecting the effect of new bubbles increase significantly.

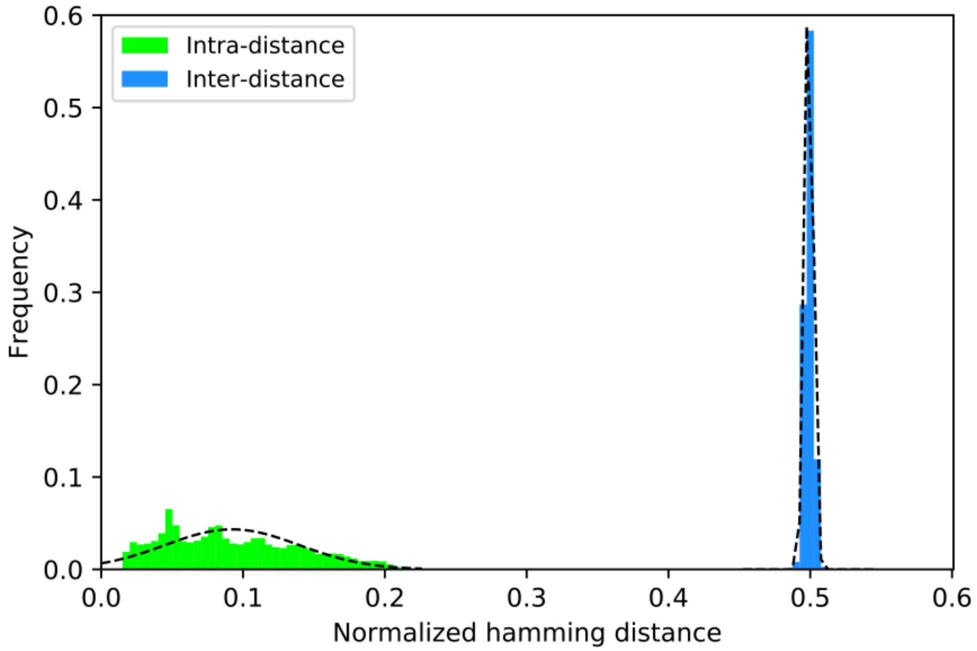

Extended Data Fig. 2. Intra and Inter distance distributions for a neutron-sensitive optical PUF. The intra-distance (reproducibility) distribution is obtained from identical challenge-response pairs for a given detector and the inter-distance distribution from comparing the responses of different detectors to a given challenge.

Extended Data Table 1. Characteristics of optically complex superheated detectors. Absorbance A data at λ = 0.635 mm was obtained with a WP-120 (Walter Product) spectrophotometer using distilled water as blank. Data are averaged over 3 different samples per detector type with the exception of the detector with no micro-spheres (referred as NS). The transmittance T, optical depth τ and transport mean free path l^* are computed from the absorbance. The cuvettes width L is 10 mm. For all detector types, the superheated drop concentration is ~4000 cm^{-3}. The micro-spheres concentration C_s is also provided.

TYPE	C_S [10^6 CM^{-3}]	A	T [%]	T	L^* [MM]
**NS**	0	0.28	52.0	0.65	15.3
**1**	8.7	1.06	8.7	2.44	4.1
**2**	17.4	1.49	3.2	3.43	2.9
**3**	34.7	1.94	1.2	4.47	2.2
**4**	52.1	2.18	0.7	5.03	2.0
**5**	69.5	2.35	0.5	5.41	1.9
